# Effectiveness and safety of allergen immunotherapy in patients with allergic rhinitis complicated by rheumatic autoimmune diseases: a case series study

**DOI:** 10.1186/s13223-022-00703-0

**Published:** 2022-07-11

**Authors:** Kazuki Fujioka, Akiko Kasahara, Takashi Kida, Wataru Fujii, Takahiro Seno, Makoto Wada, Masataka Kohno, Yutaka Kawahito

**Affiliations:** Inflammation and Immunology, Graduate School of Medical Science, Kyoto Prefectural University of Medicine, 465, Kajii-cho, Kawaramachi-Hirokoji, Kamigyo-ku, Kyoto, Japan

**Keywords:** Allergen, Allergic rhinitis, Autoimmune diseases, Contraindication, Immunotherapy

## Abstract

**Background:**

Allergen immunotherapy (AIT) is the only treatment that has modified the natural history of allergic diseases. However, since its overall effect on the immune system has not been elucidated, AIT is either absolutely or relatively contraindicated in patients with rheumatic autoimmune diseases (RADs). Therefore, there have been no long-term observations of patients with RADs receiving AIT; thus, the effectiveness and safety of AIT in these patients remain unclear.

**Methods:**

This was a single-center retrospective observational study. RAD patients receiving AIT for allergic rhinitis at our institution were selected. Changes in the activity of RAD patients were investigated for 2 years from baseline, including those who discontinued AIT. The effectiveness of AIT was also investigated using the Japan Allergic Rhinitis Standard Quality of Life Questionnaire.

**Results:**

Thirteen patients with RADs were enrolled in the study. All patients received sublingual immunotherapy, of which four discontinued AIT owing to adverse events. Among all patients, the symptoms of RADs in three patients worsened during the observation period; however, none of them were causally related to AIT. Most of the adverse events associated with AIT were mild, in which only one patient required drug intervention due to worsening rhinitis symptoms. In the nine patients who were able to continue AIT, their eye and nasal symptom scores showed a significant improvement from 1.67 (1.5–2.0) at baseline to 0.67 (0–1.17) in the 2nd year of treatment (*p* = 0.0141).

**Conclusions:**

AIT is a safe and effective treatment modality for patients with allergic rhinitis complicated by RADs.

## Introduction

Allergic rhinitis is a highly common allergic disease characterized by IgE-mediated inflammation of the upper respiratory tract [1]. In Japan, allergic rhinitis is described as a national malady. In particular, cedar pollinosis affects more than one-third of the population [2]. Allergic rhinitis not only impairs the quality of life of patients due to symptoms such as rhinorrhea, nasal obstruction, sneezing, and itching, but also causes a significant socio-economic loss due to decreased labor productivity caused by the presenteeism brought about by these symptoms [1, 3]. Therefore, overcoming allergic rhinitis has extremely important public health implications.

Currently, allergic rhinitis is mainly treated with antihistamines, leukotriene receptor antagonists, and intranasal corticosteroids [1]. Advances in these therapeutic agents have greatly improved the clinical outcome of allergic rhinitis. However, these medications are symptomatic and do not radically cure allergic diseases. Meanwhile, allergen immunotherapy (AIT), which aims to develop tolerance through the continuous administration of small amounts of allergens, is the only causative treatment that modifies the natural history of allergic diseases and can be expected to lead to a complete cure [4]. In Japan, sublingual immunotherapy for allergic rhinitis caused by cedar pollen and mites has been widely conducted [5]. However, in many guidelines, AIT was considered as an absolute or relative contraindication for patients with allergic rhinitis concomitant with rheumatic autoimmune diseases (RADs) [6, 7]. Furthermore, there are no studies that have comprehensively evaluated the impact of the biological process of AIT on the pathogenesis of RADs; there is no solid evidence that AIT does not aggravate RADs [8]. However, there are several case reports on the development of RADs after AIT, although the possibility is empirically estimated to be low [8]. On the other hand, in long-term observational studies comparing AIT and conventional anti-allergy therapy for allergic patients over 10 years, there was no significant difference in the incidence of RADs between the AIT and conventional therapy groups [9, 10]. In addition, AIT has been reported to be effective and safe in patients with allergic rhinitis complicated by acquired immune deficiency syndrome, which also causes immune abnormalities [11]. Based on these findings, AIT has gradually been extended to patients with allergic rhinitis concomitant with RADs. In fact, in an electronic survey of physicians affiliated with the European Academy of Allergy and Clinical Immunology, 29.4% answered that they had experience in administering AIT to patients with allergic diseases complicated by RADs [12].

However, it should be noted that the aforementioned safety data for AIT were estimated only in average allergic patients but not in those with RADs. Therefore, in allergic patients with RADs, the possibility that AIT negatively affects RADs cannot be excluded. Thus, it has not yet been shown whether AIT alters the activity of concomitant RADs or whether AIT has equivalent effectiveness in patients with RADs who are receiving immunosuppressive therapy. Therefore, this observational study aimed to investigate the effectiveness and safety of AIT in patients with allergic rhinitis complicated by RADs.

## Methods

### Study design

This was a single-center observational case series. We retrospectively examined the clinical data of patients with allergic rhinitis concomitant with RADs who received AIT and attended the Department of Rheumatology and Allergology at Kyoto Prefectural University of Medicine between December 2016 and December 2021. In all patients, these clinical data were examined for 2 years after the initiation of AIT, including those who discontinued AIT during treatment. The AIT protocol was performed in accordance with the manufacturers’ instructions.

### Patients

Eligible patients were defined as those aged ≥ 20 years who voluntarily expressed their intention to receive AIT. The decision on the application of AIT was not made for this study; it was made by the attending physician who judged the necessity of AIT during regular medical practice. The diagnosis and treatment of allergic rhinitis with concomitant RADs as well as the monitoring of their symptoms were performed by a physician specializing in both rheumatology and allergy.

The diagnosis of allergic rhinitis was based on the following: rhinitis symptoms during the relevant allergen dispersal period, the presence of allergen-specific IgE antibodies, or a positive reaction to the skin prick test using standardized relevant allergen extracts (Torii Pharmaceutical, Tokyo).

### Outcome measurements

The primary endpoint of this study was the proportion of patients who had worsened activity of concomitant RADs within 2 years post-AIT initiation. The clinical data used to evaluate the effect of AIT on RADs were collected at regular visits during usual medical practice.

The following indices were used to evaluate the activity of each RADs: Simplified Disease Activity Index (SDAI) for rheumatoid arthritis (RA) [13], Disease Activity Score 28-CRP (DAS28-CRP) for peripheral spondyloarthritis [14], Birmingham Vasculitis Activity Score (BVAS) for eosinophilic granulomatosis with polyangiitis (EGPA) [15], modified Rodnan total skin thickness score (m-Rodnan TSS) for systemic sclerosis (SSc) [16], serum creatine kinase (CK) level for polymyositis (PM), serum C-reactive protein (CRP) level for diffuse fasciitis, and EULAR Sjögren's syndrome disease activity index (ESSDAI) for Sjögren’s syndrome (SjS) [17].

The secondary endpoints were the effectiveness of AIT for rhinitis symptoms and the occurrence of adverse events, except for the exacerbation of RADs. Effectiveness was assessed based on the Japan Allergic Rhinitis Standard Quality of Life Questionnaire (JRQLQ No. 1) [18]. The JRQLQ was filled out by patients before the start of AIT, during the relevant allergen dispersal period in the second year of treatment for seasonal allergic rhinitis, and 2 years after the start of AIT for perennial allergic rhinitis.

The JRQLQ consists of 24 questions in eight domains: nasal and eye symptoms, usual activities, outdoor activities, social functioning, sleep problems, general physical and emotional functioning, and overall quality of life. The scores at baseline and at the second year of treatment were compared for the eight domains.

### Statistical analysis

Categorical variables were described using absolute numbers and percentages, whereas quantitative variables were described using medians and interquartile ranges. The disease activity of RADs and JRQLQ scores before and after the start of AIT were compared using the Wilcoxon signed-rank test. For all statistical tests, the significance level was set at 0.05. All statistical analyses were performed using EZR version 1.53 (Saitama Medical Center, Jichi Medical University, Saitama, Japan), which is a graphical user interface for R [19].

## Results

### Patient demographics and baseline characteristics

Thirteen patients with RADs treated with AIT were enrolled in this study. Among them, 11 had cedar pollinosis and two had perennial allergic rhinitis caused by mites. The AIT type was sublingual immunotherapy (SLIT) in all cases. The background RADs were RA in eight cases, PM with SSc in one case, SjS in one case, EGPA in one case, peripheral spondyloarthritis in one case, and diffuse fasciitis in one case. Immunosuppressive therapy for RADs was administered in 12 patients. Glucocorticoids were used in three patients; the median dose regimen was 1(0.88–2.5) mg/day of prednisolone equivalent. As immunosuppressive agents or immunomodulators, methotrexate was used in four patients (30.7%); salazosulfapyridine, bucillamine, tacrolimus, and azathioprine were also used. Biologics were used in six patients (46.1%); all of whom had RA. The details of the data are described in Table 1. Nine of the 13 patients were able to continue AIT for more than 2 years. However, four patients (three RA and one SjS) were discontinued due to the occurrence of adverse events.

**Table 1:** Patients’ demographic and baseline characteristics (n = 13)

Age (years), median (IQR)	64 (50–68)
Gender, n (%)	
Male	2 (15.4)
Female	11 (84.6)
Type of AIT, n (%)	
SLIT	13 (100)
Target of AIT, n (%)	
Cedar pollen	11 (84.6)
Mite	2 (15.4)
Concomitant asthma, n (%)	1 (7.7)
Concomitant RADs, n (%)	
RA	8 (61.5)
Other RADs	5 (38.5)
Disease duration of RADs (year), median (IQR)	7.6 (6.7–10.5)
Positive for anti-CCP antibodies, ^a^ n (%)	5 (62.5)
Positive for rheumatoid factor, ^a^ n (%)	6 (75)
Glucocorticoid use, n (%)	3 (23)
Dose(mg/day), ^b^ median (IQR)	1 (0.88–2.5)
Methotrexate use, n (%)	4 (30.7)
Dose(mg/day), ^b^ median (IQR)	6 (5.75–6.5)
Other immunosuppressants use, n (%)	2 (15.3)
Immunomodulators use, n (%)	2 (15.3)
Biologics use, n (%)	6 (46.1)

Other RADs consisted of 1 case of polymyositis with systemic sclerosis, 1 case of Sjogren’s syndrome, 1 case of eosinophilic granulomatosis with polyangiitis, 1 case of peripheral spondyloarthritis, and 1 case of diffuse fasciitis without eosinophilia

The other immunosuppressants were tacrolimus and azathioprine. Immunomodulators were sulfasalazine and bucillamine. Biologics were golimumab, abatacept, and tocilizumab

AIT, Allergen immunotherapy; SLIT, sublingual immunotherapy; RA, Rheumatoid arthritis; RADs, Rheumatic autoimmune diseases; CCP, cyclic citrullinated peptide

^a^The rates of rheumatoid factor and anti CCP antibody were calculated for the population of rheumatoid arthritis patients

^b^Median glucocorticoid and methotrexate dose were calculated among each agent users

### Clinical course of RADs

**Fig. 1:**
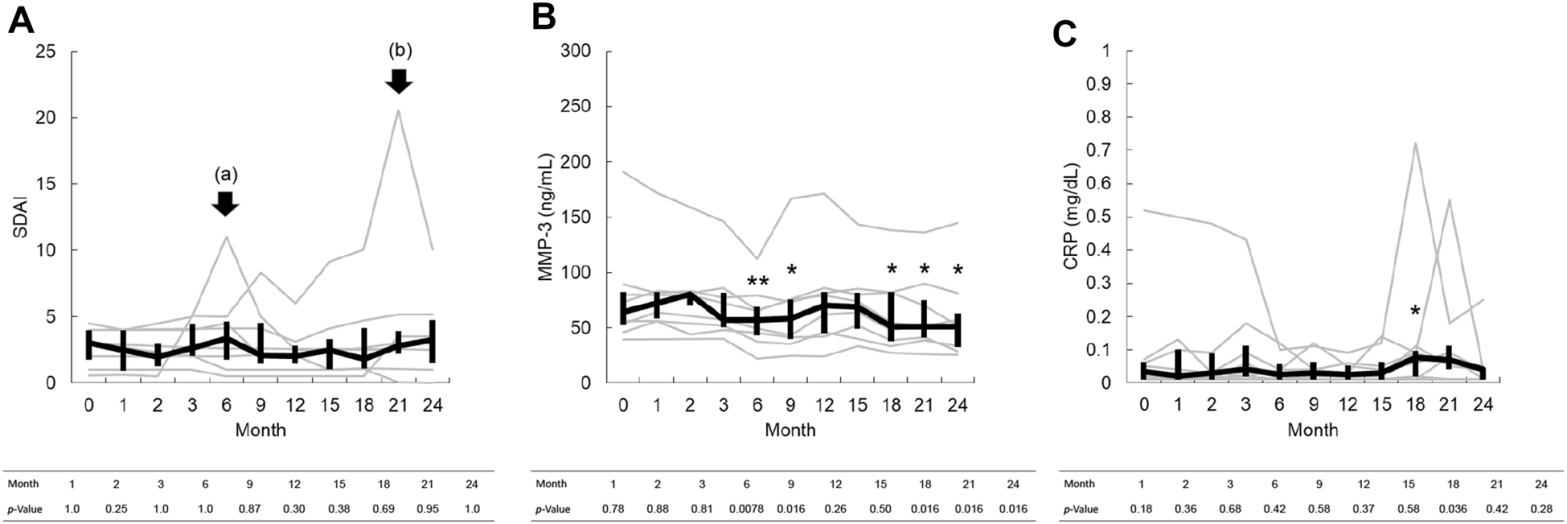
Changes in rheumatoid arthritis-related parameters after the initiation of allergen immunotherapy (AIT). **A** simplified disease activity score (SDAI). **B** matrix metaroproteinase-3 (MMP-3). **C** c-reactive protein (CRP). For all parameters, comparisons were made between baseline and each of the following time points with the Wilcoxon signed rank test: 1 month, 2 months, 3 months, 6 months, 9 months, 12 months, 15 months, 18 months, 21 months, and 24 months. Each gray line corresponds to an individual value; the median and interquartile ranges are indicated by bold black lines. Two patients had worsening disease activity, which is indicated by the arrows

The baseline disease activities of all eight RA patients were under 11.0 on the SDAI score, thereby indicating remission or low disease activity. There was no significant worsening of the SDAI score or matrix metalloproteinase-3 (MMP-3) values compared to baseline at all timepoints within 2 years post-AIT initiation, including cases of AIT discontinuation (Fig. 1A, B). Only the CRP level worsened significantly from 0.035 (0.01–0.0625) at baseline to 0.075 (0.0175–0.095) within 18 months. However, both were within normal limits and the change was not clinically meaningful (Fig. 1C). However, two patients showed an elevation in the SDAI score during the observation period. In the first case, although the SDAI score deteriorated during AIT, there were no obvious inflammatory findings on blood tests or joint ultrasonography; the symptoms spontaneously improved without any intensified treatment. Therefore, it was concluded that the symptoms were caused by temporary environmental stress and thus did not change the activity of RA (Fig. 1A, arrow a). The second case was also judged to have deteriorated due to factors other than AIT; the patient had discontinued AIT 1 week post-initiation. Furthermore, the deterioration may have occurred immediately after extending the administration interval of the biological agent (Fig. 1A, arrow b).

**Fig. 2:**
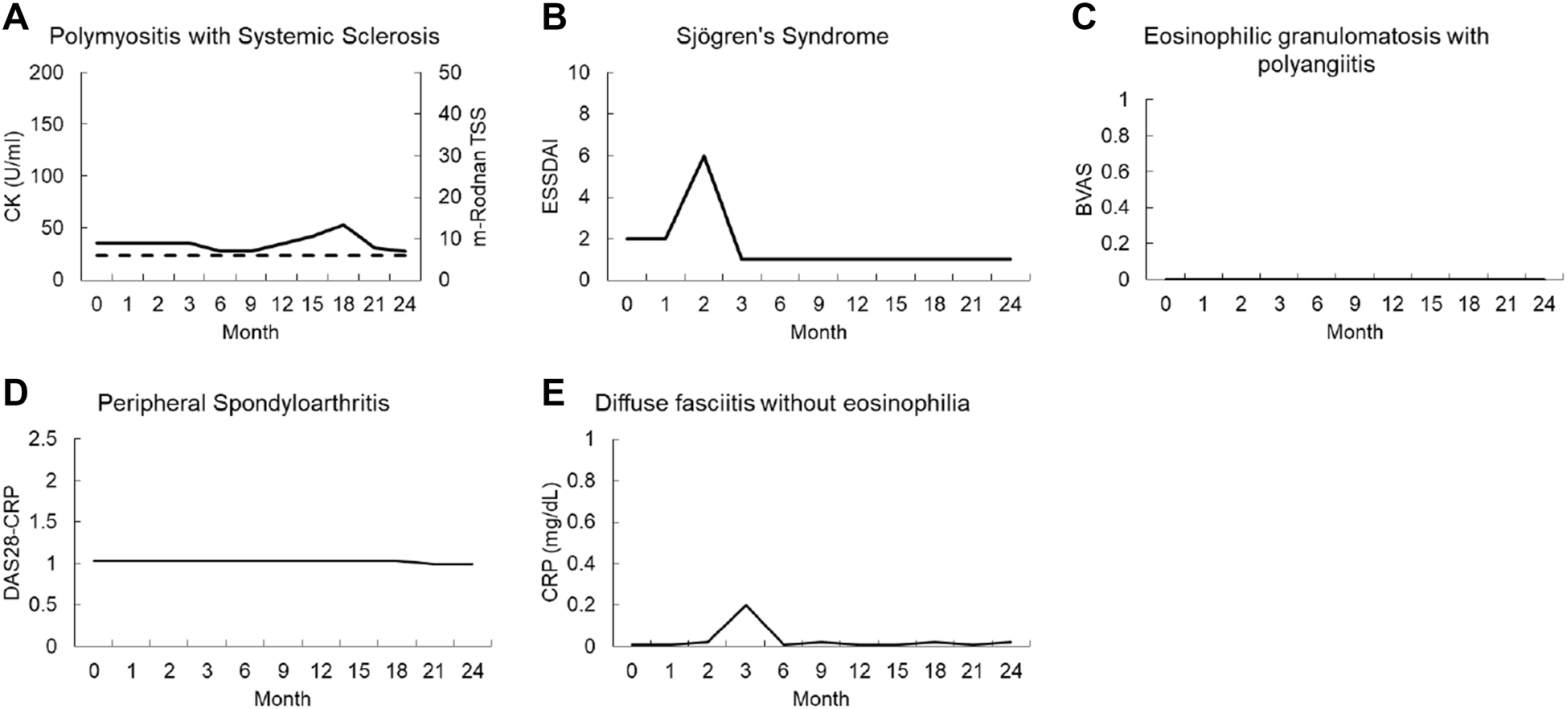
Changes in activity assessment parameters of rheumatic autoimmune diseases other than rheumatoid arthritis after initiation of allergen immunotherapy (AIT). **A** Polymyositis with systemic sclerosis. Black and broken lines represent creatinine kinase (CK) value and modified Rodnan total skin score (m-Rodnan TSS), respectively. **B** Sjögren's syndrome. **C** Eosinophilic granulomatosis with polyangiitis. **D** Peripheral spondyloarthritis. **E** Diffuse fasciitis without eosinophilia. ESSDAI, EULAR Sjögren's syndrome disease activity index; BVAS, Birmingham vasculitis activity score; DAS28, disease activity score 28; CRP, C-reactive protein

The other patients with RADs did not show any change in disease activity during the observation period, except for one patient with Sjogren's syndrome who experienced a temporary worsening of disease activity (lymphadenopathy). However, it was judged that there was no causal relationship between AIT and lymphadenopathy in this case because of the following reasons: AIT was discontinued a few days after the start of AIT, the timing of the appearance of lymphadenopathy was only 2 months later, and similar symptoms had appeared before the start of AIT. The course of these cases is shown in the Fig. 2.

### Safety profile

During the observation period, 11 adverse events were considered to be related to AIT (Table 2). There were no serious adverse events of grade 3 or higher according to the grade classification of Common Terminology Criteria for Adverse Events (CTCAE—version 5.0); most of them were mild [20]. Only one case (7.7%) of nasal discharge requiring antihistamine administration was observed as a moderate adverse effect. Although there was a case of asthma complication, in which asthma symptoms worsened during AIT, it was only transient and mild, thus not requiring therapeutic intervention and not leading to the discontinuation of AIT. The most common adverse reaction was oral pruritus in three patients (23.1%); it was not severe. However, AIT was self-discontinued in four patients due to these adverse events; three discontinued within 1 week and the other discontinued at 6 months after initiation due to oral pruritus or laryngeal discomfort.

**Table 2:** JRQLQ scores at baseline and second year of treatment (n = 9)

	Baseline	2nd year	p value
Nasal and eye symptoms	1.67 (1.5–2.0)	0.67 (0–1.17) *	0.0141
QOL related questionnaires			
Usual daily activities	1.0 (0.8–1.4)	0 (0–0.8)	0.0575
Outdoor activities	1.0 (0.5–2.0)	0 (0–0.5) *	0.0350
Social functioning	0.67 (0.33–1.0)	0 (0–0)	0.584
Impaired sleeping	2.0 (1.0–2.0)	0 (0–1.0)	0.0655
Physical problems	1.0 (1.0–2.0)	0 (0–1.0)	0.114
Emotional functions	1.0 (0.5–1.5)	0 (0–0)	0.106
General state	4.0 (3.8–4.0)	2.0 (1.0–2.0) *	0.0498

The data are presented as median with interquartile ranges

Comparison of each JRQLQ score between baseline and second year were calculated using the Wilcoxon signed rank test. *p < 0.05

### Effectiveness for rhinitis

In nine patients (five with RA, one with PM and SSc, one with EGPA, one with peripheral SpA, and one with diffuse fasciitis) who were able to continue AIT for more than 2 years, allergic rhinitis symptoms showed significant improvement after the start of AIT as evaluated by the JRQLQ questionnaire. The median score for eye and nose symptoms improved from 1.67 (1.5–2.0) at baseline to 0.67 (0–1.17) at the second year of treatment, thus showing a statistically significant difference. In QOL-related items, the score for outdoor activity improved from 1.0 (0.5–2.0) at baseline to 0 (0–0.5) at the second year of treatment, thus also showing a statistically significant difference. There were no statistically significant differences in the other domain scores; however, there was a downward trend in all domains. The overall index of the general state also improved from 4.0 (3.8–4.0) at baseline to 2.0 (1.0–2.0) at the second year of treatment, thus showing a statistically significant difference. Changes in each QOL-related item are presented in Table 3.

**Table 3:** Adverse events during AIT other than worsening of RADs (n = 13)

	n (%)
Oral pruritus	3(23.1)
Throat irritation	2(15.4)
Nasal discharge	2(15.4)
Sneeze	1(7.7)
Nausea	1(7.7)
Abdominal discomfort	1(7.7)
Dyspnea	1(7.7)

Each value represents the number of patients

## Discussion

This was a two-year observational study investigating whether AIT affects the activity of concomitant RADs and whether AIT is effective in patients with allergic rhinitis complicated by RADs. As far as studies evaluating the impact of AIT on underlying RADs were concerned, there was only one case report showing that AIT was safe for RA after a 1-year observation [21]. The reason for the lack of studies was speculated to be that allergists are generally reluctant to perform AIT in patients with allergic rhinitis complicated by RADs [6]. Certainly, there are several reports on the appearance of autoimmune diseases such as rheumatoid arthritis [22], systemic lupus erythematosus [23], Sjogren’s syndrome [24], and vasculitis after AIT [25, 26].

The reason for the development of these RADs is not clear. It was speculated that some of these reports were relatively old and the allergen extracts used for AIT were not well-purified, which might lead to the formation of immune complexes and the development of the disease [8]. The quality of allergen extracts has been improving due to the progress of purification and standardization technologies, thereby indicating that this risk may have been relatively reduced in recent years [27]. In this study, many patients with RADs were able to continue AIT and did not experience worsening of RAD activity. Only three patients experienced a temporary worsening of RAD activity; however, their causal relationship to AIT was denied by the attending physician, either because AIT had already been discontinued or because of other clear-cut factors. In addition, most adverse events other than those related to RADs were minimal, thereby suggesting that AIT may be performed without major problems, even in patients with RADs.

In general, AITs may have an unfavorable effect on RADs because of their mechanism. Although not fully understood, it is mainly due to the suppression of the Th2-type immune response by Treg induction [28], allergen-specific immune deviation from Th2 to Th1 represented by interferon-γ (IFN-γ) production [29, 30], and antigen-specific IgG4 antibody production [31]. Of these, Th1-type cytokines, such as IFN-γ, have been reported to contribute to the pathogenesis of RADs. A number of reports suggest that IFN-γ-producing CD4 + T cells function as effector cells at the site of inflammation in RA [32]. A meta-analysis of microarray data from RA patients has shown that IFN-γ is a potent upstream regulator of RA synovial biology [33]. In systemic lupus erythematosus, IFN-γ signaling promotes germinal center responses and the production of autoreactive B cells, thereby leading to disease progression [34]. In fact, some patients treated with IFN-γ have been reported to develop lupus-like symptoms [35, 36]. Therefore, the possibility of RAD deterioration under IFN-γ-induced conditions cannot be ruled out. However, although IFN-γ is used as a treatment for renal cancer, idiopathic pulmonary fibrosis, and chronic granulomatous diseases, it has not been reported that autoimmune diseases occur frequently in these trials [37–39]. In this study, worsening of RADs due to AIT was not observed. Although caution should be exercised regarding the worsening of symptoms when AIT is administered to patients with autoimmune diseases, it is presumed that this possibility is low.

On the other hand, the present study also investigates the effectiveness of AIT. The symptoms and QOL scores in the second year of treatment showed significant improvement compared to baseline. Even if statistically significant differences were not detected, most scores decreased. In a phase III trial evaluating the efficacy of SLIT in cedar pollinosis, the improvement in the general state index of JRQLQ was about 20% in the 2nd year compared with the placebo; the result of this study was not inferior to this result, even though direct comparison was difficult [40]. Since AIT has been reported to improve treatment outcomes with the passage of time, further improvements in efficacy can be expected in the future [41].

In this study, most of the enrolled patients were using glucocorticoids, immunosuppressive agents, or biologics; thus, there was a concern that these drugs might impair the effectiveness of AIT. Furthermore, the immunogenicity of the vaccine has been reported to decrease when administered to patients with RADs receiving immunosuppressive therapies, such as glucocorticoids, methotrexate, rituximab, and abatacept [42]. On the other hand, it has been reported that short-term glucocorticoids or anti-cytokine therapy targeting TNF-α and IL-6 do not impair the immunogenicity of the vaccine [43, 44]. Additionally, the negative effects of glucocorticoids and methotrexate on immunogenicity were noted to be dose-dependent [45]. This study enrolled patients who used these drugs at low doses, which might explain why the effectiveness of AIT did not disappear. It is also possible that the immunogenicity of AIT does not completely disappear even if it is affected; thus, the effect of AIT may have appeared.

This study has several limitations. First, this was a small, single-center, retrospective study that could not eliminate unintentional selection bias. Most of the concomitant RADs had low activity levels; patients with moderately or highly active RADs were not included. If these patients had been treated with AIT, it is possible that their outcomes would have been different. Second, we could not observe the details of the immunological effects of AIT due to the lack of immunological data, such as Treg and antigen-specific IgG4, as well as rheumatoid factor and anti-CCP antibody trends. Third, to accurately evaluate the safety and effectiveness of AIT in patients with RADs, it is desirable to compare the results from allergic rhinitis patients without RADs who underwent AIT. Therefore, the results of this study should be validated in a prospective multicenter study using such allergic rhinitis patients as controls.

In conclusion, our results suggest that AIT is safe and effective in patients with allergic rhinitis complicated by RADs with low activity level. AIT is expected to become a potential therapeutic option for patients with allergic rhinitis complicated by RADs who have failed to achieve satisfactory improvement with conventional pharmacotherapy.

## Data Availability

All data generated or analyzed during this study are included in this published article.

## Abbreviations

AIT: Allergen immunotherapy
RADs: Rheumatic autoimmune diseases
RA: Rheumatoid arthritis
SDAI: Simplified disease activity index
DAS28: Disease activity score 28
BVAS: Birmingham Vasculitis Activity Score
EGPA: Eosinophilic granulomatosis with polyangiitis
m-Rodnan TSS: Modified Rodnan total skin thickness score
SSc: Systemic sclerosis
PM: Polymyositis
ESSDAI: EULAR Sjögren's Syndrome Disease Activity Index
SjS: Sjogren’s syndrome
JRQLQ: Japan allergic rhinitis standard quality of life questionnaire
MMP-3: Matrix metalloproteinase-3
CCP: Cyclic citrullinated peptide
CK: Creatine kinase
CRP: C-reactive protein
